# In Memoriam: Laurentiu Mircea Popescu (1944–2015)

**DOI:** 10.1111/jcmm.12688

**Published:** 2015-09-04

**Authors:** Xiangdong Wang

**Affiliations:** a Zhongshan Hospital Biomedical Research Center, Shanghai Institute of Clinical Bioinformatics, Fudan UniversityShanghai, China; b King’s College of LondonLondon, UK

**Figure:**
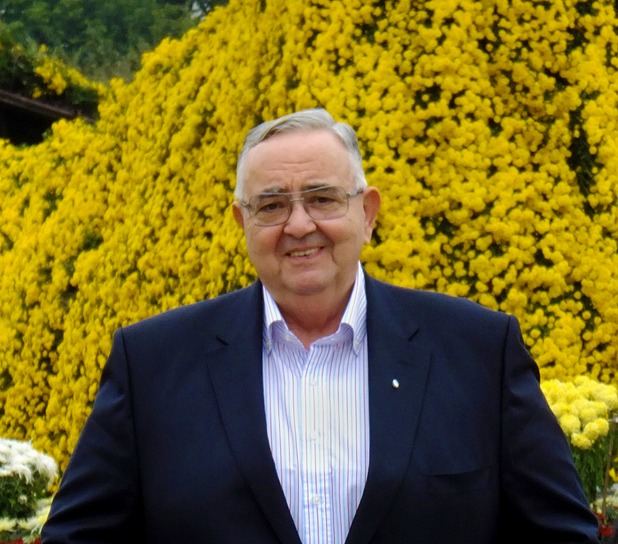


The world of cellular and molecular medicine is deeply saddened and shocked by the tragic news that Professor Laurentiu Mircea Popescu has passed away due to lung cancer on Monday morning of August 3, 2015. Professor Popescu was a world-recognized leader of cellular and molecular medicine and worked in the University of Medicine and Pharmacy, Bucharest, since 1991 before becoming the General Director of ‘Victor Babeș’ National Institute of Pathology, Fellow of the Romanian Academy of Sciences, and President of the Medical Sciences Section of the Romanian Academy of Sciences. He was selected as vice-President of the Federation of the European Academies of Medicine during 2010–2011 and became the President in 2012. He was also the founding editor-in-chief of the Journal of Cellular and Molecular Medicine.

‘The book has to be written by scientists and it has to tell the world of the true cellular and molecular story about telocytes. Telocytes are connecting cells, while we are connecting people’ wrote Professor Popescu. I could not believe that these were the last words I got weeks ago from Prof Popescu, a science colleague, life friend, career supervisor, and respected leader. Professor Popescu and I have been planning to write a book on telocytes for almost 2 years and finally decided ‘Telocytes: Connecting Cells’ as the name of the book in the beginning of 2015. All of the authors feel the honor and responsibility in writing their own chapters in memory of Prof Popescu who discovered and coined the term ‘telocytes’ in 2010. Professor Popescu provided us with leadership and his global and deep vision on telocyte science inspired and encouraged all of us. He was labeled as the father of telocytes, and leaves behind a large number of remarkable milestones and legacies, especially on telocytes.

Professor Faussone-Pellegrini, as a pioneer scientist of the interstitial cell of Cajal, was impressed by Professor Popescu’s enthusiasm in the research field of telocytes and remembered that she was contacted and convinced to initially collaborate on the interstitial cell of Cajal. After they published a number of papers where Professor Popescu named those cells as interstitial Cajal-like cells, Professor Popescu suddenly appeared at her laboratory and told her that the name of interstitial Cajal-like cells needed to be reconsidered. After a long discussion with colleagues, Professor Popescu decided to think out of the box and preferred to call the term ‘telocyte’ on the basis of the suggested name ‘teledendrocyte: tele = far away, dendro =ramifications/branches/processes, cyte = cell’. Among different opinions and suggestions, Professor Popescu showed his leadership capacity and insisted on the name of telocyte as a term easier to remember/use. Professors Popescu and Faussone-Pellegrini published a milestone editorial titled ‘TELOCYTES - a case of serendipity: the winding way from interstitial cells of Cajal, *via* interstitial Cajal-like cells to TELOCYTES’ in the Journal of Cellular and Molecular Medicine in 2010 and officially announced the start of a telocyte era. After that, Professor Popescu took global leading roles in telocyte research and shared his knowledge and experience with scientists around the world.

His vision in the field of telocytes and cellular and molecular medicine is legendary. He was one of the first to realize that telocytes were located in multi-organs/tissues, to propose the existence of ‘Telocytes/Stem-Cells Tandem’ working in so-called ‘Stem Cell Niches’, and to identify three-dimensional structures and morphology of telocytes. The Telocyte concept is well accepted by hundreds of scientists from more than 60 universities located in 30 countries. I started the investigation of lung telocytes in the middle of 2010 and wrote to Professor Popescu for an ‘urgent help’ when I thought the mission to be impossible. With direct supervision of Professor Popescu, telocytes were firstly isolated from human trachea and lungs in our laboratory and cultured in an *in vitro* system. In order to identify the difference of pulmonary telocytes from other resident cells within lungs, we collaborated with Professor Popescu’s group to investigate genomic and proteomic profiles of telocytes and compare them with mesenchymal stem cells, fibroblasts, alveolar type II cells, airway basal cells, proximal airway cells, CD8^+^ T cells from bronchial lymph nodes, and CD8^+^ T cells from lungs.

His global leading role was also based upon his knowledgeable vision. He is the first man to propose that telocytes may interact with stem cells since he found that telocytes are often located along with stem cells. The hypothesis was afterward evidenced by a number of scientific articles. In August, 2015, just before Professor Popescu left us, he showed new evidence that secretomes of myocardial telocytes could modulate the activity of cardiac stem cells. His group found that telocytes interact with cardiac stem cells *via* paracrine effects of telocytes-produced extracellular vesicles. They characterized telocyte secretomes, studied protein secretory profiles, and compared secretome profiles of telocytes with stem cells. Three major portals of elements in telocyte secretomes include growth factors, chemoattractants, and cytokines/chemokines, indicating that telocytes may regulate stem cell growth and differentiation, cell recruitments, or microenvironmental formations. Professor Popescu believed that the interaction between telocytes and stem cells could be the principle of new therapies for severe diseases. Building on Professor Popescu’s idea, we have designed a number of experimental studies to find out the optimal delivery of co-transplanted stem cells and telocytes, evaluated dynamic lung distributions and retentions of transplanted cells at dynamic durations after intravenous, intratracheal, or intraperitoneal deliveries of stem cells or telocytes alone or in combination after induction of acute lung injury. Without sharing those data and visions, telocyte research would not be possible or would not have reached its current success.

Professor Popescu spent almost half a decade of his life to investigate and promote cellular and molecular medicine in order to share his vision, knowledge, and experience. He started the Journal of Cellular and Molecular Medicine, initiated the mechanism investigation of cGMP- induced relaxation in vascular smooth muscle, and received a number of medals and rewards, including the Gold Medal of Paris Academy from ‘Rene Descartes’ University, the Merit Medal from International Society for Heart Research, the Gold Medal from ‘Albert Schweitzer’ International Academy, and the Gold Medal from International Academy of Cardiovascular Sciences. Professor Popescu is one of the few foreign experts to win the ‘Magnolia Award’ which is the highest honor of the Shanghai Municipal People’s government for recognition of Shanghai’s economic construction and social development made by outstanding contributions of expatriates which has a 20-year history. Professor Popescu chaired a large number of international meetings and was the leader of various professional societies. Professor Popescu was a man, an example, and an enthusiastic drive to inspire scientists and young generations, to spread the light for the development of other people, to help anyone as needed, and most importantly make a difference.

‘I hope I can be the light that guides all of you’ said Professor Popescu to the cell and molecular biologists when I co-chaired with him in a telocyte meeting. From the start he realized the importance of developing a common, world-wide accepted education to benefit young scientists. He spent valuable time on each graduate student and young doctor and commented on their work and manuscript word by word. ‘He is an extremely serious professor when we are talking about experimental results, an amiable gentleman when we discuss about life and living, and a respected supervisor when we go through the manuscript’, Dr. Yonghua Zheng, whose PhD thesis was also supervised by Professor Popescu, felt. Professor Popescu was the man that inspired young scientists to become thinkers, creators, and explorers of new sciences. He motivated people to work on and publish articles with high standards, establish stable methodologies, apply new biotechnologies, develop new research program, and make us more visible and active. ‘I did not work directly on telocytes, but I knew Professor Popescu well. He visited me in Louisville 4 years ago. It was so sad that scientific community lost such great man… Nevertheless his work will stay alive forever’, said Dr. Mariusz Z. Ratajczak who is a Professor and the Endowed Chair, Director of Stem Cell Program, University of Louisville, USA.

‘Telocytes need the world’, was emphasized by Professor Popescu who realized that the whole world need to be involved in order to be successful in the endeavor of telocyte research. He supported the active participation of scientists, professors, principle investigators, and students from all over the world in all initiatives and promoted special sessions for platform presentations to present the successes achieved as well as the specific challenges faced in the telocyte world. Without Professor Popescu’s leadership, generosity of spirit, and persistent optimism in joining people, efforts, and achievements together, we can only wonder where we would be in understanding of telocytes and the delivery of telocytes-associated therapies.

I know many others would have been better positioned to write this memoriam of Professor Popescu since many of you had been working with Professor Popescu much closer and longer, received more energetic inspirations from Professor Popescu, made much more successful achievements under Professor Popescu’s supervisions, or had a much closer relationship with Professor Popescu. One thing we will all agree on is that there are no words that can express our sadness and deep condolences at this very moment. Please do join me to thank him for his persistence and powerful drive for all of us to improve telocyte research. You and I are all enriched for knowing and working with Professor Popescu and I urge the consideration of the Laurentiu Mircea Popescu Foundation to ensure that his example continues to inspire us all and keep him alive in our hearts.

